# Particularities of the implant-supported prosthesis in patients with bruxism: systematic review of the literature

**DOI:** 10.11604/pamj.2026.53.93.47329

**Published:** 2026-02-20

**Authors:** Ismail Chawki, Imane Ihoume, Akram Leghtas, Meriem Amine

**Affiliations:** 1 Fixed Prosthesis Department, Faculty of Dentistry of Casablanca, Hassan II University of Casablanca, Casablanca's Dental Consultation and Treatment Center, CHU Ibn Rochd, B.P 9157, Mers Sultan, Casablanca, Morocco

**Keywords:** Dental implant, dental prosthesis, bruxism, implant-supported, occlusal overload

## Abstract

Bruxism concerns diurnal and nocturnal parafunctional activities, including grinding, rubbing, tapping, and clenching of the teeth. This study aims to assess the longevity and reliability of implant-supported prostheses made for patients with bruxism and establish the criteria for success and failure for these prostheses. A search in the Medline-PubMed, Web of Science, Scopus, and Google Scholar databases was conducted; the last computer search was on 20^th^ October 2025, with no period filter. Studies published in English with a follow-up period of at least 6 months were considered. The writing of this literature review followed the guidelines of the PRISMA Statement (Preferred Reporting Items for Systematic Reviews and Meta-Analysis). Implant survival rates among bruxers ranged from 57.5% to 100% over an average time period of 12 to 291 months, remaining lower than those noted in non-bruxers. Rates of mechanical complications reach up to 60%, and prosthetic failure rates up to 29.3% in patients with bruxism. However, wearing a protective splint was associated with a 1.8-fold reduction in wear of prosthetic materials. The particularities found in patients with bruxism require the practitioner to take certain precautions during the rehabilitation with implant supported prosthesis to be able to make up for its vulnerability and ensure its sustainability.

## Introduction

Bruxism is a complex parafunction that corresponds to a repetitive and unconscious activity of the masticatory muscles. It affects between 8 and 31.4% of the population [1,2]. It can present 2 circadian manifestations distinctly or combined: Daytime bruxism: while awake, characterized by clenching of teeth, where contractions are generally involuntary and of variable intensity/duration [3]. Nocturnal bruxism: during sleep, characterized by clenching and grinding of the teeth [4]. From an etiological perspective, awake bruxism seems more directly associated with psychological traits [5,6], whereas sleep bruxism is a complex activity with multiple neurological implications and interactions with other sleep-related conditions [7,8]. The dental clinician is the first witness to the manifestations of this parafunction, including dental wear, periodontal damage, and dysfunctions of the orofacial musculature and temporo-mandibular joint (TMJ) [9].

Patients with bruxism may require prosthetic rehabilitation during their overall care. It is generally accepted that, when it comes to tooth-supported prostheses, bruxism would be incriminated in the occurrence of complications or even therapeutic failure [10]. This parafunction could complicate the treatment plan and affect the prognosis of the prosthetic restoration, particularly when it comes to implant-supported prosthetic rehabilitation [11]. So, how does bruxism impact the success and prognosis of implant-supported prostheses? The objective of this systematic review of the literature is to evaluate the longevity and reliability of implant-supported prostheses made in patients with bruxism, and to establish the success and failure criteria for these prostheses.

## Methods

**Redaction protocol:** this systematic review of the literature was carried out in accordance with the recommendations of The PRISMA Statement “Preferred Reporting Items for Systematic reviews and Meta-Analyses” [12].

**Figure 1:**
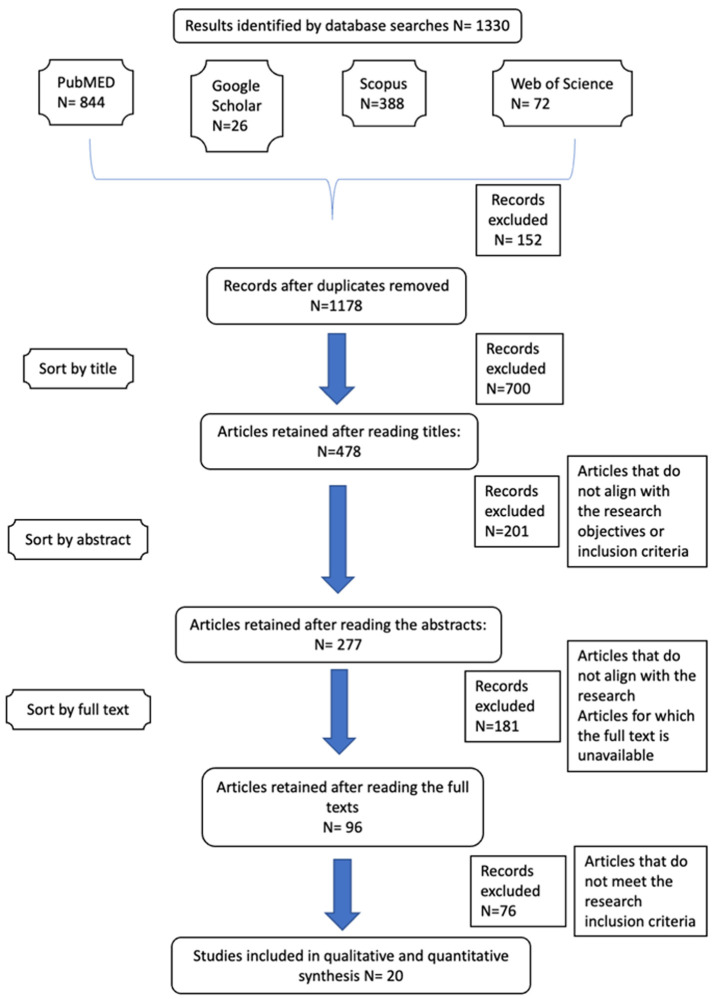
flow diagram for literature screening and selection

**Research strategy:** an electronic search was conducted using keywords with Boolean equations. The literature search was based on English-language electronic databases accessible via the Internet. The databases used were: Medline-PubMed, Web of Science, Scopus, and Google Scholar. The Boolean equations chosen for the electronic search were: dental implant and (bruxism or bruxers or parafunctional or clenching or grinding or occlusal overload), dental prosthesis, implant-supported, and (bruxism or bruxers or parafunctional or clenching or grinding or occlusal overload). The date of the last computer search was 20/06/2025, with no period filter. The search strategy is shown in the PRISMA flow diagram in Figure 1.

### Eligibility criteria

**Inclusion criteria:** the articles selected in our literature search are clinical studies that met the following inclusion criteria: i) evaluating the role of bruxism as a risk factor in implant-supported prosthetic rehabilitations; ii) conducted on human subjects; iii) having a follow-up duration of at least 6 months; iv) written in English.

**Exclusion criteria:** the articles excluded from our computerized search were as follows: i) case series; ii) expert opinions; iii) studies conducted on animals or cadavers; iv) in vitro and in silico experiments; v) literature reviews and meta-analyses.

**Selection of literature:** the reading of the scientific articles was carried out by two independent readers, A.L and M.A. A first reading was carried out to select the articles through the title and the abstract, following the eligibility criteria. Then a second full reading of the selected articles was carried out, eliminating articles that do not meet the inclusion criteria. In the event of disagreement between the opinions of the two independent readers, a discussion made it possible to resolve the differences and find a common consensus.

**Data extraction:** to prepare and structure our systematic review, the targeted question was developed using the PICO criteria [13]: P: participants ⇒ patients with bruxism; I: intervention ⇒ placement of dental implants; C: comparison ⇒ success and complication rates between the bruxer and non-bruxer groups; O: “outcomes” ⇒ impact of bruxism on the implant-supported prosthesis. The success criteria published by Albrektsson *et al*. [14]. were adopted, whereas implant survival was defined as implants that remained in situ at the time of the most recent follow-up appointment with no indication for removal. Complications in implant therapy comprise two categories: biological and technical (mechanical) [15]. Biological complications refer to reactions in the peri-implant hard and soft tissues, while technical ones are related to mechanical damage of the implant/implant components and superstructures. Variables such as the evolution over time of the parameters cited above and the statistical differences (p) between each intervention group in each study were studied.

**Quality appraisal of included studies:** the critical reading of the articles relied on methodological quality assessment tools specific to the types of studies included in our literature review:

**Qualitative assessment of non-randomized studies:** the evaluation of non-randomized clinical studies was based on the Newcastle-Ottawa Scale, which was developed by the universities of Newcastle in Australia and Ottawa in Canada [16]. The tool for assessing the risk of bias of non-randomized studies from the “Cochrane Handbook for Systematic Reviews of Interventions,” which provides advice to authors for the preparation of their systematic reviews [17]. Using the Newcastle-Ottawa Scale (NOS), to facilitate consistent interpretation, studies were further categorized as good, fair, or poor quality based on the thresholds defined by the Agency for Healthcare Research and Quality (AHRQ). Studies were rated good if they scored 3 or 4 stars in the Selection domain, 1 or 2 stars in Comparability, AND 2 or 3 stars in the Outcome/Exposure domain; fair if they scored 2 stars in Selection, 1 or 2 in Comparability, AND 2 or 3 in Outcome/Exposure; and poor if they scored 0 or 1 in Selection, OR 0 in Comparability, OR 0 or 1 in Outcome/Exposure [18].

The risk of bias assessment using the tool recommended by the Cochrane Handbook for Systematic Reviews of Interventions ('The Handbook') was carried out for each article. We examined the following biases: confounding bias, selection bias, bias in the classification of interventions, bias due to deviation from intended interventions, attrition bias, reporting bias, bias in outcome measurement, and other biases [17].

## Results

**Description of search results:** the literature searches yielded a total of 1330 potentially relevant titles and abstracts (Figure 1). Preliminary exclusion was performed for duplicate references; after reading the titles and abstracts, 277 articles were included for full-text presentation. After meeting the inclusion and exclusion criteria, a total of 20 studies were included for qualitative analysis. The selected articles included 6 prospective cohorts and 14 retrospective cohorts, with a follow-up period ranging from 6 months to 36 years. The 20 reviewed studies included a total of 9313 patients and a total of 31209 implants.

### Bruxism as a failure factor of the implant-supported rehabilitation: survival rates and complications of the implant-supported rehabilitation in bruxers

Ten selected studies [19-28] focused on the impact of bruxism on implant and prosthetic survival rates, as well as the rates of occurrence of mechanical and biological complications on the implant-supported prosthesis, and compared these rates to those found in nonbruxer patients, to be able to conclude (Table 1, Table 1.1). In this literature review, the examined studies reported implant survival rates ranging from 57.5% [28] to 100% [29-31], and prosthetic survival rates ranging from 70.6% [32] to 100% [29,33,34] for a follow-up of 12 to 291 months. Following the evaluation of these non-randomized clinical studies using the NEWCASTLE-OTTAWA scale (Table 2), they were categorized as good (7 studies), fair (1 study), and poor quality (2 studies). The assessment of bias using the Cochrane Handbook for Systematic Reviews of Interventions, “The Handbook” (Table 3), shows that most articles present an uncertain risk for bias in the measurement of outcomes, because no information is given on the status of the evaluators. Some articles also present a high bias risk in the selection of participants.

**Table 1:** PICO criteria for assessing the impact of bruxism on implant survival and complication rates

Authors	Study design	Participants	Interventions	Comparison/parameters studied	Results
Chrcanovic BR *et al*. 2020	Retrospective cohort	642 patients rehabilitated with 876 plural fixed prostheses on implants of 2 to 6 prosthetic units, out of a total of 2241 implants; 289 men and 353 women average age of 57.8 years (14.5-90.9); bruxism diagnosis through self-report and clinical examination	Clinical and radiological evaluation of treatment results	Group 1: 79 prostheses in patients with bruxism (48 of whom wear a night splint); group 2: 610 prostheses in nonbruxers; mechanical complication rate	108 months follow-up; implant failure rate: 5.7% among bruxers and 4% nonbruxers; no significant difference P=0.279, prostheses: 88 failures (10%), with a significantly higher prevalence among bruxers (19%) P=0.005
Chatzopoulos GS *et al*. 2020	Retrospective study	2127 patients with 4519 implants; average age of 59.57 ± 13.83 years (18-93); 1055 men 1072 women; 25.4% have self-reported bruxism	Clinical and radiographic evaluation from patient records to determine implant survival rates	Implant survival rate	Average follow-up: 34 months; Implant failure rate: 1.7%; the association between bruxism and implant failure shows no statistical significance (P=0.881)
Chitumalla R *et al*. 2018	Retrospective cohort	450 patients; 240 men, 210 women; carriers of 640 implants; bruxism diagnosis through self-report and clinical examination	Clinical and radiographic assessment of the presence of complications	Control group: 326 nonbruxers; test group: 124 patients with bruxism; mechanical complications	Follow-up: 5 years; implant survival rate In men bruxers: 1 year: 90%/5 years: 72; in women: 1 year: 92%/5 years: 70%. Complication occurrence rate by prosthesis type: single crown: 29%, partial prosthesis: 37%, complete prosthesis: 37% (P=0.012)
Chrcanovic B R *et al*. 2018	Retrospective study	2670 patients with 10099 implants supporting prosthetic restorations of different types; bruxism diagnosis through self-report and clinical examination	Clinical and radiological evaluation in search of implant fractures	Group 1: 6761 implants in nonbruxers; group 2: 446 implants in patients with bruxism; implant fracture rate	Follow-up from 34.6 months to 155.5 months; implant failure (fracture) rate: among bruxers 3.6% and among nonbruxers, 0.3%; bruxers had an increased probability of 1819.5% of presenting an implant fracture compared to nonbruxers
Mikeli A *et al*. 2016	Retrospective study	144 patients with 507 implant prostheses; 69 patients (47.9%) diagnosed with bruxism through self-report and clinical examination, 9 of whom use an occlusal splint	Clinical examination of prostheses looking for fractures, cracks, and wear facets on the ceramic, then classify into 4 groups according to the severity of the damage	Control group: 75 nonbruxers; test group: 69 patients with bruxism; ceramic fracture	Average follow-up: 5 years; prosthetic failure rate: 34.8% bruxers and 13.3% nonbruxers; bruxism is a risk factor for ceramic fracture of fixed implant prostheses (P=0.002)

**Table 1.1:** PICO criteria for assessing the impact of bruxism on implant survival and complication rate

Chrcanovic BR *et al*. 2016	Retrospective cohort	994 patients with 3549 implants; 478 men with 1777 implants; 516 women with 1772 implants; bruxism diagnosis through self-report and clinical examination	Clinical and radiological evaluation of implant survival	Control group: 938 patients nonbruxers; test group: 56 patients with bruxism; implant survival rate	Average follow-up: 95 months; implant failure rate among bruxers 13%, among nonbruxers 4.6%; bruxism is a statistically significant risk factor for implant failure (P=0.012)
Chrcanovic BR *et al*. 2017	Retrospective cohort	186 patients; 854 implants; 98 bruxers diagnosed through self-report and clinical examination; 98 nonbruxers; each group includes: 49 men; 49 women; 427 implants	Clinical and radiographic evaluation and consultation of archives for survival rate and mechanical complications	Control group: 98 patients are nonbruxers; test group: 98 patients with bruxism (59 use a nighttime splint); mechanical complications Implant survival rate.	Average follow-up: 115 months; implant failure rate among bruxers 25.5% compared to 11.2% among nonbruxers (P=0.001); a higher prevalence of mechanical complications in bruxers (P=0.001): ceramic fracture; loosening and unscrewing; screw fracture.
Kinsel RP *et al*. 2009	Retrospective cohort	152 partially or totally edentulous patients with 729 implants supporting: 390 single crowns; 94 fixed plural prostheses; 67 men (44.1%); 85 women (55.9%). Bruxism diagnosis: either self-reported or clinical signs of occlusal wear patterns	Clinical and radiological evaluation	Ceramic fracture rate group 1: 43 patients (312 prosthetic units) with bruxism; group 2: 109 nonbruxers (686 prosthetic units)	6-month follow-up: patient-level prosthetic failure rate: among bruxers, 34.9% compared to 17.2% among nonbruxers; bruxism is significantly associated with a higher rate of ceramic fracture on implant-based prostheses (P = 0.01); protective effect of the splint
Brägger U *et al*. 2001	Retrospective cohort	85 partially edentulous patients 53 women; 32 men average age of 55.7 years (23-83); 10 bruxers; no clear criteria for bruxism diagnosis	Installation of a total of 116 plural fixed prostheses (of 3 types: on implant; on dental support; and combined; on a total of 105 implants and 144 dental abutments	Group I-I (implant-supported prosthesis: 33 patients, 40 prostheses, 84 implants; Group I-T (tooth-implant-supported prosthesis): 15 patients, 18 prostheses, 19 implants, and 18 teeth; group t-t (tooth-supported prosthesis): 40 patients, 58 prostheses, 124 teeth	Follow-up: 4 to 5 years: loss of one prosthesis from each group (i-i, i-t, t-t) -2 implants lost by fracture; biological complications were not significantly associated with patient risk factors; 6/10 patients with bruxism suffered mechanical complications (60%), compared to 13/75 without bruxism (17.3%), P=0.01; presence of a correlation between bruxism and mechanical complications
Chrchanovic BR *et al*. 2018	Retrospective study	221 patients with 1045 implants; 95 men (405 implants) and 132 women (640 implants); delayed loading; no clear criteria for bruxism diagnosis	Clinical and radiographic evaluation to analyze treatment results.	Group 1: 87 implants in patients with bruxism; group 2: 662 implants in nonbruxers; implant survival rate; marginal bone loss rate.	Average follow-up: 291 months; cumulative survival rate of 87.8% after 36 years of follow-up; among bruxers: 42.53% of implant failures compared to 11.93% among nonbruxers, P=0.001; Bruxism negatively affects implant survival rate, due to implant fractures and marginal bone loss

**Table 2:** Newcastle-Ottawa scale evaluation of selected articles

Authors	Final score	Score interpretation
Chrcanovic BR *et al*. 2020	Selection: 1+1+1+1	Good quality
	Comparability: 1+1	
	Outcome: 1+1+1	
Chatzopoulos GS *et al*. 2020	Selection: 1+0+0+1	Fair quality
	Comparability: 1+1	
	Outcome: 1+1+1	
Chitumalla R *et al*. 2018	Selection: 1+0+1+1	Poor quality
	Comparability: 0+0	
	Outcome: 1+1+0	
Chrcanovic BR *et al*. 2018	Selection: 1+1+1+1	Good quality
	Comparability: 1+1	
	Outcome: 1+1+1	
Mikeli A *et al*. 2016	Selection: 1+1+1+1	Good quality
	Comparability: 1+0	
	Outcome: 1+1+0	
Chrcanovic BR *et al*. 2016	Selection: 1+1+1+1	Good quality
	Comparability: 1+1	
	Outcome: 1+1+1	
Chrcanovic BR *et al*. 2017	Selection: 1+1+1+1	Good quality
	Comparability: 1+1	
	Outcome: 1+1+1	
Kinsel RP *et al*. 2009	Selection: 1+1+0+0	Poor quality
	Comparability: 1+0	
	Outcome: 1+0+0	
Brägger U *et al*. 2001	Selection: 1+1+0+1	Good quality
	Comparability: 1+0	
	Outcome: 1+1+1	
Chrchanovic BR *et al*. 2018	Selection: 1+0+1+1	Good quality
	Comparability: 1+1	
	Outcome: 1+1+1	
De Angelis F *et al*. 2017	Selection: 1+1+1+1	Good quality
	Comparability: 1+0	
	Outcome: 1+1+1	
Papi P *et al*. 2017	Selection: 1+1+1+1	Good quality
	Comparability: 1+0	
	Outcome: 1+1+0	
Ibañez JC *et al*. 2005	Selection: 1+1+1+1	Good quality
	Comparability: 1+1	
	Outcome: 1+1+0	
Ji Ting-Jen *et al*. 2012	Selection: 1+1+1+1	Good quality
	Comparability: 1+1	
	Outcome: 1+1+1	
Glauser R *et al*. 2001	Selection: 1+1+1+1	Good quality
	Comparability: 1+0	
	Outcome: 1+1+1	
Coltro *et al*. 2018	Selection: 1+1+1+1	Good quality
	Comparability: 1+1	
	Outcome: 1+1+0	
Chrcanovic BR *et al*. 2020	Selection: 1+1+1+1	Good quality
	Comparability: 1+1	
	Outcome: 1+1+1	
Engstrand P *et al*. 2003	Selection: 1+1+1+1	Good quality
	Comparability: 1+1	
	Outcome: 1+1+1	
Mangano FG *et al*. 2013	Selection: 1+1+1+1	Good quality
	Comparability: 1+0	
	Outcome: 1+1+1	
Koenig et Wulfman *et al*. 2019	Selection: 1+0+1+1	Good quality
	Comparability: 1+0	
	Outcome: 1+1+1	

**Table 3:** estimation of the risk of bias of the included studies

Author and year of study	Confounding bias	Selection of participants bias	Bias in the classification of interventions	Deviation bias from intended interventions	Attrition bias (missing data)	Reporting bias (selection of the reported results)	Bias in the measurement of outcomes	Other biases
Chrcanovic BR *et al*. 2020	Minimal	Minimal	Minimal	Minimal	Minimal	Minimal	Uncertain	Minimal
Chatzopoulos GS *et al*. 2020	Minimal	High	Minimal	Minimal	Minimal	Minimal	Uncertain	Minimal
Chitumalla R *et al*. 2018	Minimal	Minimal	Minimal	Minimal	Minimal	Minimal	Uncertain	Minimal
Chrcanovic B R *et al*. 2018	Minimal	Minimal	Minimal	Minimal	Minimal	Minimal	Uncertain	Minimal
Mikeli A *et al*. 2016	Minimal	Minimal	Minimal	Minimal	Minimal	Minimal	Uncertain	Minimal
Chrcanovic B R *et al*. 2016	Minimal	Minimal	Minimal	Minimal	Minimal	Minimal	Uncertain	Minimal
Chrcanovic B R *et al*. 2017	Minimal	High	Minimal	Minimal	Minimal	Minimal	Uncertain	Minimal
Kinsel R P *et al*. 2009	Minimal	Minimal	Minimal	Minimal	Minimal	Minimal	Minimal	Minimal
Brägger U *et al*. 2001	Minimal	High	Minimal	Minimal	Minimal	High	Uncertain	Minimal
Chrchanovic B R *et al*. 2018	Minimal	Uncertain	Minimal	Minimal	Minimal	High	Uncertain	Minimal
De Angelis F *et al*. 2017	Minimal	Uncertain	Minimal	Minimal	Minimal	Minimal	Minimal	Minimal
Papi P *et al*. 2017	High	Uncertain	High	Minimal	Uncertain	Uncertain	Minimal	Minimal
Ibañez J C *et al*. 2005	Minimal	Minimal	Minimal	Minimal	Minimal	Minimal	Uncertain	Minimal
Ji Ting-Jen *et al*. 2012	Minimal	Minimal	Minimal	Minimal	Minimal	Minimal	Uncertain	Minimal
Glauser R *et al*. 2001	Minimal	Minimal	Minimal	Minimal	Minimal	Minimal	Uncertain	Minimal
Coltro *et al*. 2018	Uncertain	Minimal	Minimal	Minimal	Minimal	Minimal	Minimal	Minimal
Chrcanovic BR *et al*. 2020	Minimal	Minimal	Minimal	Minimal	Minimal	Minimal	Uncertain	Minimal
Engstrand P *et al*. 2003	Minimal	High	Minimal	Minimal	Minimal	Minimal	Uncertain	Minimal
Mangano FG *et al*. 2013	Minimal	Minimal	Minimal	Minimal	Minimal	Minimal	Minimal	Minimal
Koenig V *et al*. 2019	Minimal	High	Minimal	Minimal	Minimal	Minimal	Minimal	Minimal

**Bruxism as a risk factor for implant failure:** among the selected studies, 2 looked at the impact of bruxism on implant survival rates, compared to other known risk factors, such as smoking, grafted sites, poor prosthetic designs, crown-to-implant ratio (c/i)<0.8, and the abutment angulation>25° (Table 4). Long-term clinical and radiological monitoring of implants in the group of patients suffering from bruxism made it possible to establish failure rates of 12.93% and 15% in the studies of De Angelis *et al*. in 2017 [35] and Papi *et al*. in 2017 [36], respectively. The association of bruxism and risky loading (c/i ratio < 0.8; angulation > 25°; presence of cantilever) gives the highest rates of mechanical and biological failure (69.23% success rate) [35]. According to the Newcastle-Ottawa Scale (Table 2), both studies are of good quality. Both De Angelis *et al*. in 2017 [35] and Papi *et al*. in 2017 [36] studies present an unclear to high risk for bias in the selection of participants, in the selection of the reported results, and in the measurement of outcomes (Table 3).

**Table 4:** PICO criteria addressing bruxism as a risk factor for implant failure

Authors	Study design	Participants	Interventions	Comparison/parameters studied	Results
De Angelis F *et al*. 2017	Retrospective cohort	225 patients with 871 implants; average age 55.6 (18-79); 145 men (64.44%) and 80 women (35.56%); no clear criteria for bruxism diagnosis	Retrospective evaluation and long-term clinical and radiological follow-up of the effects of risk factors on implant failure	Group A: 33 patients with bruxism/116 implants group B: 24 smoking patients/85 implants; group C: 15 patients who received a bone graft/30 implant; group D: 18 patients with risky load (c/i ratio < 0.8; angulation > 25°; presence of cantilever)/58 implants; implant survival rate	Average follow-up of 13.6 years (10-18); in Group A, 15/116 implant failures (success rate = 89.66%), 3/116 implants lost (survival rate = 97.41%); seen separately, bruxism presents the highest rate of implant failure, while the combination of bruxism and lateral loading presents the highest mechanical and biological failure rates
Papi P *et al*. 2017	Retrospective cohort	89 patients; 56 men; 33 women average age of 53.24 (23-76 years); carriers of 227 implants Having at least 1 of the following mechanical risk factors: bruxism, c/i (crown-to-implant) ratio less than 0.8, abutment angulation; bruxism diagnosed through clinical examination	Clinical and radiological evaluation of implant survival	Group 1: 152 implants in patients with bruxism; group 2: 45 in patients with the c/i ratio less than 0.8; group 3: 30 implants had an abutment angulated by more than 25 degrees; implant survival rate	Average follow-up: 13.6 years (10-16 years); failure rate for each group: group 1: 15% (P≤0.05)/group 2: 11% (P≥0.05)/group 3: 10% (P≥0.05); bruxism was the only variable to show a statistically significant association with implant failure (P<0.05)

### Therapeutic specificities of the implant-supported restorations in patients with bruxism

**Immediate loading:** three selected studies: Ibañez *et al*. 2005 [29], Ji *et al*. 2012 [33], and Glauser *et al*. 2001 [37], focused on immediate loading in patients with bruxism, and concluded implant failure rates of 0.97%, 29.3% for fixed total implant-supported prostheses, and 41% for all types of implant-supported prostheses (Table 5, Table 5.1). According to the Newcastle-Ottawa Scale (Table 2), all 3 studies are of good quality. These studies present an uncertain risk of bias in the measurement of outcomes (Table 3).

**Table 5:** PICO criteria addressing immediate implant loading, total edentulism, and posterior restorations in patients with bruxism

Authors	Study design	Participants	Interventions	Comparison/ parameters studied	Results
Ibañez JC *et al*. 2005	Prospective cohort	41 total mono or bi maxillary edentulous patients; average age of 62.1 years (38-82) 30 women and 11 men; bruxism is diagnosed through self-report	Placement of 343 implants; immediate loading; 217 in the maxilla, 126 in the mandible; 207 implants in patients with bruxism; Prosthetic restoration with 49 fixed total prostheses on an implant	Group A: 12 restorations with immediate loading of a temporary fixed resin prosthesis; group B: 10 restorations with a temporary resin prosthesis reinforced with metal, 4 to 24 hours after implant placement; group C: 27 immediate loading of the definitive prosthesis, 48 hours following implant placement; implant survival rate	Follow-up: 6 years; cumulative success rate of 99.42%; success rate in patients with bruxism (99.03%) compared to 100% for non-bruxers; these results suggest that immediate loading, in totally edentulous patients, is not contraindicated by bruxism
Ji Ting-Jen *et al*. 2012	Retrospective study	45 patients with a total of 297 implants immediately loaded with 50 fixed total prostheses on implants; 18 men/27 women average age 61.5 years (25-88); 58 implants in bruxers; no clear criteria for bruxism diagnosis	Clinical and radiographic evaluation of treatment results; immediate loading	Implant survival rate; marginal bone loss rate; prosthetic complication	Followed by an average of 42.1 months; 269/297 implants remained in function, cumulative success rate of 85.2%, an absolute success rate of 90.6%; a higher failure rate in patients with bruxism, with 17/58 (29.3%) compared to 11/239 (4.6) in nonbruxers
Glauser R *et al*. 2001	Prospective study	41 patients; 19 men; 22 women; average age of 52 years (19-72); no clear criteria for bruxism diagnosis	Placement of 127 implants; 22 implants in patients with bruxism; immediate loading (at the latest 11 days after implant placement); 58 prosthetic restorations (unitary, partial, and total)	Implant survival rate	Follow-up: 1 year; a cumulative success rate of 82.7% after one year; Failure rate in patients with bruxism (41%) versus (12%) in those without bruxism (P=0.002); significant association between implant failure and bruxism, during immediate loading
Coltro *et al*. 2018	Prospective cohort	88 patients with 29 men; 59 women; average age of 64.9 94 screw-retained, metal-acrylic IFCDs (implant-supported complete dentures); bruxism diagnosis through self-report, followed by BiteStrip® device measurements	Placement of screw-retained, metal-acrylic IFCDs; Clinical evaluation of the implants and prosthesis	Prosthetic survival and success rates; rate of mechanical complications	Average follow-up of: 2.93 years; prosthesis survival rate: 100%; prosthesis success rate: 83%; mechanical complications: 17% (mainly artificial teeth fracture/loosening); bruxism: Not significantly associated (p > 0.05); framework design: significant risk factor (<4 mm pins): HR 11.038, (p= 0.027)

**Table 5.1:** PICO criteria addressing immediate implant loading, total edentulism, and posterior restorations in patients with bruxism

Chrcanovic B R *et al*. 2020	Retrospective study	709 uni- or bimaxillary total edentulous patients, having received 869 total fixed prostheses on implants, for a total of 4797 implants 318 men and 391 women; average age of 64 years (20.7-90); bruxism diagnosis through self-report and clinical examination	Clinical and radiological evaluation of treatment results.	Group 1: 51 prostheses in bruxers; group 2: 557 prostheses in nonbruxers; prosthetic and implant survival rate; rate of mechanical complications	Average follow-up of 10.7 years; bruxers showed higher implant (25.35% vs 5.6%; p < .001) and prosthetic failure rates (29.4% vs 6.3%; p = .001) than non-bruxers; significant association of bruxism with increased rates of loosening (HR: 3.302), screw fracture (HR: 4.956), ceramic crack/fracture (HR: 5.685), resin tooth fracture (HR: 2.125).
Engstrand P *et al*. 2003	Prospective cohort	95 total edentulous patients in the mandible: 53 men (56%); 42 women (44%); average age of 68.5 years; 5 patients with bruxism; no clear criteria for bruxism diagnosis	Placement of 285 implants using the Brånemark Novum technique prosthetic rehabilitation: fixed total prosthesis on an implant in the mandible.	Implant survival rate; marginal bone loss rate	Average follow-up of 2.5 years (1-5); 18 implants were lost 6.3%, implant survival rate of 93.3% over 5 years; a single lost prosthesis; 2/5 (40%) bruxers suffered implant failure compared to 11/90 (12.2%) non-bruxers, P=0.27.
Mangano FG *et al*. 2013	Prospective cohort	194 patients (104 men and 90 women); average age of 49.1 years (24-74); 35 (18%) smokers and 24 (12.3%) bruxers; bruxism diagnosis through self-report, clinical and instrumental diagnosis (electromyography)	Placement of 215 implants (locking taper) 8 mm length; single crown support in the posterior region; delayed loading 124 (57.7%) implants in the maxilla, and 91 (42.3%) in the mandible	Implant and prosthetic survival rate; marginal bone loss rate; mechanical complications; biological complications	Followed by an average of 5.6 years (1-10); 3 lost implants; Implant survival rate for bruxers (95.5%) and nonbruxers (98.8%), P=0.266; 2 biological and 3 prosthetic complications out of the 215 implant crowns; prosthetic survival rate: bruxers (87.7%) and nonbruxers (96.9%) P=0.052; differences not statistically significant.
Koenig et Wulfman *et al*. 2019	Prospective cohort	45 patients; 61.7% affected by bruxism; 19.1% wear a night guard; bruxism diagnosis through self-report and clinical examination	Placement of 75 second-generation monolithic 3Y-TZP zirconia posterior restorations corresponding to 101 elements; on 85 implants and 10 dental abutments; 20.3% were plural restorations.	Prosthetic survival rate; mechanical complications	Follow-up: 2 years; the restorations had a survival rate of 93.3% ± 2.9% and a success rate of 81.8% ± 4,7%; 80% of catastrophic failures and 76.9% of all complications occurred in patients with bruxism; survival rate in nonbruxers 95.7 ± 4.3% vs 92.3% ± 3.7% among bruxers P=0.6; success rate among nonbruxers 87% ± 7% vs 79.6% ± 5.9% among bruxers P=0.55. Statistically insignificant difference

### Impact of bruxism depending on the type of edentulism

**Bruxism and total edentulism:** the studies by Chrcanovic *et al*. 2020 [32], Engstrand *et al*. 2003 [34], and Coltro *et al*. in 2018 [31], which investigated the impact of bruxism in total edentulous patients rehabilitated with an implant-supported prosthesis, report implant survival rates ranging from 74.65% to 100%, and prosthetic survival rates ranging from 70.6% to 100% (Table 5, Table 5.1). According to the Newcastle-Ottawa Scale (Table 2), all studies are of good quality. The studies by Chrcanovic *et al*. in 2020 [32] and Engstrand *et al*. in 2003 [34] present an uncertain risk of bias in the measurement of outcomes, and a high risk of bias in the selection of participants. The Coltro *et al*. study in 2018 showed a moderate risk of bias due to confounding (Table 3)[31].

**Bruxism and posterior edentulism:** two studies focused on the impact of bruxism in patients with posterior edentulism rehabilitated by the implant-supported prosthesis [30,38] (Table 5, Table 5.1); Koenig *et al*. in 2019 [38], show that the prosthetic success rate is 79.6% and the prosthetic survival rate is 92.3%, Mangano *et al*. in 2013 [30] reported a prosthetic survival rate of 87.7%. According to the Newcastle-Ottawa Scale (Table 2), both studies are of good quality. The Koenig *et al*. in 2019 [38] study presents a high risk of bias in the selection of participants (Table 3).

## Discussion

Bruxism has been very strongly associated with mechanical/prosthetic complications, with prevalences far exceeding those observed in nonbruxers, for different forms of complications, notably unscrewing and loosening, fracture of the abutment, of the screw, or the implant, deformation of the implant and the prosthesis, and fracture of the ceramic [21,23,25-27,32,38].

Although the majority of the included studies were observational in design, and despite heterogeneity in clinical protocols, follow-up durations, and bruxism diagnostic criteria, the convergence of findings across a large number of implants supports the role of bruxism as a relevant risk factor for implant and prosthetic failure. The methodological quality assessment using the Newcastle-Ottawa Scale indicated that most included studies were of good methodological quality, with only a limited number rated as fair or poor [20,21,26]. Evaluation using the ROBINS-I tool, as recommended by the Cochrane Handbook, identified domain-specific limitations inherent to non-randomized designs, predominantly related to outcome measurement and participant selection, while confounding bias was limited in most studies. Taken together, these findings suggest that heterogeneity and bias may affect the precision of effect estimates, but do not undermine the consistency or clinical relevance of the observed associations.

In the specific context of investigating bruxism, randomized controlled trials assigning patients to a potentially harmful exposure are neither ethically acceptable nor practically feasible. Consequently, retrospective and prospective cohort studies represent the most appropriate methodological framework for evaluating this association. Nevertheless, RCTs may indirectly support the clinical relevance of bruxism-related risks by assessing whether interventions aimed at mitigating parafunctional loading, such as occlusal splint therapy, influence implant-related outcomes. The parafunctional activity of bruxism generates excessive forces of the order of 50 kgf [39], and for a period that exceeds the 20 minutes of interdental contact normally observed in a day during chewing and swallowing, where occlusal forces average only 26.7 kgf and 30.2 kgf, respectively [40,41].

According to studies by Chrcanovic *et al*. in 2020 [22] and Mikeli *et al*. in 2016 [23], the chances of these complications appearing in bruxers are up to 18.19 times more frequent for implant fractures, and 3.6 times higher for ceramic fractures than for non-bruxers. It is considered that these results are partly related to the reduced proprioception of implants compared to natural teeth. Indeed, the periodontal ligament of natural teeth provides the central nervous system with feedback for sensory perception and motor control. The periodontal sensitivity threshold is less than 1 N for the anterior teeth and around 4N for the posterior teeth [42]. while the threshold for perception of information transmitted by an osseointegrated implant is around 6.7N [43], and information of the protopathic, vague, diffuse type. Proprioception around dental implants is limited due to the absence of a periodontal ligament, which results in less sensitivity and consequently limits the proprioceptive feedback mechanisms to the muscles [44]. In addition, the periodontal ligament has a protective role in shock absorption and stress distribution, thus preserving the integrity of the tooth-bone structure. In the case of implant-supported dentures, impact testing has revealed that implants can transmit greater forces to the surrounding bone than natural teeth, which can increase stress and the risk of complications [45].

**Recommendations:** in view of current data from the literature, it is undeniable that bruxism constitutes a significant risk factor for implant and prosthetic failure [35,36]. Consequently, treatment with the implant-supported prostheses in patients with bruxism must take into consideration a certain number of recommendations and precautions:

**Implant planning:** the objective would be to avoid elements placed in extension, and to reduce or eliminate occlusal contacts during lateral movements, for this the ideal would be to place 1 implant for each tooth to be replaced [46,47]. The axis of application of forces must be that of the long axis of the implant and the alveolar crest, in order to limit shearing and bending forces [48].

**Implant materials:** fragility of implant materials can increase the susceptibility to fracture found in bruxers; it is therefore strongly recommended not to place zirconia (zirconium dioxide) implants in these patients. On the other hand, the material of choice is TiCp grade 4, which is a titanium alloy richer in O2 and more mechanically resistant [49].

**Surface condition:** retentive threads and sanded and/or etched micro-textured surface conditions represent the solution of choice, due to their high wettability, thus promoting the covering of this surface by blood, allowing better osseointegration of the implant [50].

**Implant dimensions:** the more the diameter of the implant increases, the more the stress exerted on the cortical bone will decrease. The use of large implants, while maintaining a minimum thickness of bone around them, is therefore recommended, since they make it possible to create a larger support zone, and reduce tensions on the level of the peri-implant bone [51].

**Prosthetic design:** the prostheses must be designed with the aim of improving the distribution of stress on the implants; the implants must be installed perpendicular to the curves of Spee and Wilson to favor the direct contacts generated during the vertical function on the long axis of the implants. Most authors agree that prosthetic rehabilitation should provide a single point of contact close to the center of the implant, whenever possible. Occlusion should be marked by gently sloped cusps and small occlusal tables to protect the implant system against transverse components of forces during tooth grinding [46,52].

**Loading:** with single-tooth or partial edentulism, there are more failures in the case of immediate loading (within 2 weeks following installation), or early (6 to 8 weeks after installation) [53]. However, implant-supported total prostheses prove to be an exception, since the stress is distributed over all the implants, thus improving the survival rate [32,34]. For the bruxing patient, delayed loading is recommended, ranging from 6/10 weeks up to 1 year according to the authors, in order to ensure that the osseointegration of the implants has taken place, and thus to avoid early solicitations [11]. Sarmento *et al*. in 2012 [46], state that delayed-loaded implants have the best chance of survival, followed by immediately-loaded implants (up to 2 weeks after placement), with early-loaded implants (6 to 8 weeks after placement) being those with a lower survival rate.

**Abutment and connection systems:** the connection systems recommended for bruxers have been poorly studied according to the literature. The implant abutments should ideally be straight; they will be screwed when dealing with a Morse cone, and screwed if it is an external or internal polygon [46].

**Prosthetic materials:** the use of ceramic is therefore inevitable in bruxers, but it is, however, recommended that there be a metal infrastructure to rigidify the prosthesis, especially to a large extent [47,49]. The best compromise for a patient suffering from bruxism would therefore be a ceramic-metallic prosthesis with systematic wearing of a protective splint, in order to intercept problems of fragility of the ceramic [23].

**Splinting:** the splinting of crowns and bridges is recommended since it allows better distribution of occlusal forces, thus reducing tensions at the bone level. Fortin *et al*. concluded through a study in 2016 that the splinting of prostheses would not have an impact on peri-implant cratering, but that it would avoid contact point problems, which can, in the event of a hiatus, be the cause of inflammation and therefore possible cratering [54].

**Implant retention method:** screw-retained prostheses have the advantage of being an easily removable system, which is practical, particularly when it is necessary to manage complications or carry out prosthetic repair.

**Protective splint:** Kinsel *et al*. 2009 [26]. and Papaspyridakos *et al*. 2019 [55] agree on the protective effect of the nighttime splint, particularly in bruxers. It has been proven that the risk of wear of prosthetic materials was 1.8 times higher in patients without a splint compared to patients regularly wearing a protective splint [26,55]. The use of the splint is therefore systematically recommended in patients with this parafunction, since it helps to alleviate muscular tension and reduce the stress exerted on the prostheses. The protective occlusal splint should provide uniform and simultaneous occlusal contacts in centric relation, allowing optimal distribution of masticatory forces and prevention of complications [49]. Hard splints are preferred, as they minimize the damage to the oral tissues, promote more even occlusal load distribution, and redirect clenching and grinding forces toward a vertical axis, unlike soft splints, which may increase muscular activity [46].

During mandibular movements, the splint should ensure posterior disocclusion in protrusion and strictly anterior contacts during lateral excursions. In cases of single or partial implant-supported prostheses, when a sufficient number of natural teeth are present to bear occlusal loads, the splint should be relieved over implant restorations [46]. Occlusal contacts must be harmonious, well-distributed, and simultaneous to prevent occlusal trauma, highlighting the importance of occlusal equilibration. To avoid undesirable effects such as extrusion or intrusion, the splint should cover all teeth, including third molars, while eliminating premature contacts [3]. The maxilla is the preferred arch for splint therapy due to its greater exposure to eccentric parafunctional forces, increased mobility of maxillary teeth, and higher susceptibility to fracture [3].

**Regular maintenance:** routine maintenance of implant-supported prostheses is crucial, particularly in patients with bruxism, where elevated occlusal forces increase the risk of mechanical complications. Annual maintenance visits should include structured occlusal checks, clinical and radiographic evaluation of implant stability and prosthetic integrity, and monitoring of peri-implant tissues. Specific attention should be paid to the detection of screw loosening, ceramic chipping, wear of prosthetic materials, and changes in the occlusal scheme. In bruxers, the presence and condition of protective night splints must be verified and adjusted if needed. Regular maintenance and occlusal adjustments significantly contribute to reducing complication rates and prolonging the longevity of implant restorations [56].

## Conclusion

Bruxism is a risk factor for implant failure. Bruxers present particularities that negatively impact the success and durability of implant-supported prosthetic restorations. However, respecting the recommendations cited above, which include wearing a protective splint, ensuring adequate monitoring and maintenance and occlusal equilibration respecting the chosen occlusal concept, enhances the prognosis.
